# Expired Patents

**Published:** 1907-08

**Authors:** 


### PATENTS.

### EXPIRED PATENTS.

June 24, 1907.

430639. Artificial Denture. N. J. Goodwin, Hartford, Conn.
430849. Surgical Forceps. F. W. Groth, Chicago, Ill.
431020. Dental Engine Motive Gear. P. Brown, Montreal, Quebec, Canada.

July 8, 1907.

431713. Dental Polishing Pencil. K. C. Whaley, Pomeroy, Ohio.
431849. Dental Articulator. J. W. Moffitt, Phila., Pa.

The above lists of patents are furnished by Davis & Davis, solicitors of American and foreign patents, Washington, D. C., and St. Paul Building, New York City.

